# Enhancing Process Control and Quality in Amorphous Solid Dispersions Using In-Line UV–Vis Monitoring of L* as a Real-Time Response

**DOI:** 10.3390/pharmaceutics17020151

**Published:** 2025-01-23

**Authors:** Mariana Bezerra, Juan Almeida, Matheus de Castro, Martin Grootveld, Walkiria Schlindwein

**Affiliations:** 1 GlaxoSmithKline, David Jack Centre, Harris Lane, Ware SG12 0GX, UK; 2 Applied Materials, Daresbury WA4 4AB, UK; juan_almeida@amat.com; 3 Leicester School of Pharmacy, De Montfort University, Leicester LE1 9BH, UK; p2827923@my365.dmu.ac.uk (M.d.C.); mgrootveld@dmu.ac.uk (M.G.)

**Keywords:** quality by design (QbD), amorphous solid dispersions (ASD), piroxicam, Kollidon^®^ VA64, hot melt extrusion (HME), in-line UV–Vis spectroscopy, principal component analysis (PCA)

## Abstract

**Background:** This study demonstrates the application of the sequential design of experiments (DoE) approach within the quality by design (QbD) framework to optimize extrusion processes through screening, optimization, and robustness testing. **Methods:** An in-line UV–Vis process analytical technology (PAT) system was successfully employed to monitor critical quality attributes (CQAs) of piroxicam amorphous solid dispersion (ASD) extrusion products, specifically lightness (L*). **Results:** L* measurement proved highly effective for ensuring the quality and uniformity of ASDs, offering real-time insights into their physical appearance and process stability. Small variations in L* acted as early indicators of processing issues, such as phase separation or bubble formation, enabling timely intervention. This straightforward and rapid technique supports real-time process monitoring and control, allowing automated adjustments to maintain product consistency and quality. By adopting this strategy, manufacturers can minimize variability, reduce waste, and ensure adherence to quality target product profiles (QTPPs). **Conclusions:** Overall, this study highlights the value of in-line UV–Vis spectroscopy as a PAT tool in hot melt extrusion, enhancing CQA assessment and advancing the efficiency and reliability of ASD manufacturing.

## 1. Introduction

Over the past two decades, regulatory authorities have collaborated with industry to advance changes in the quality paradigm, aiming to understand pharmaceutical processes and product quality more comprehensively. This new approach, often referred to as the ’enhanced’ approach or pharmaceutical quality by design (QbD), encourages innovation across the pharmaceutical lifecycle [1,2,3,4]. The goal of pharmaceutical development is to design quality products and manufacturing processes that consistently meet patient needs. This begins with a quality target product profile (QTPP), which outlines the intended use, dosage, delivery, and attributes like dissolution, stability, and appearance. Development involves iterative formulation and process design, balancing prior knowledge with experimentation, guided by risk assessment. Adopting QbD at the research and development stage helps streamline development by focusing on what is necessary, using risk-based approaches, leveraging prior knowledge, and avoiding unexpected issues. This reduces development time and facilitates regulatory discussions. At the manufacturing stage, QbD enhances product robustness, lowers costs, improves quality, and allows internal flexibility for changes.

A major challenge in the development of new chemical entities is their poor aqueous solubility [5,6,7]. To address this, various formulation strategies have been developed, such as co-crystals and inclusion complexes [8,9,10,11,12], which aim to improve the solubility of active pharmaceutical ingredients (APIs). Solid dispersion manufacturing, in particular, is of significant interest in pharmaceutical science research [13,14,15,16]. The biopharmaceutical classification system (BCS) class II and IV drugs are ideal candidates for solid dispersion technology, which enhances their solubility and overcomes bioavailability limitations. The bioavailability of these solid dispersions depends on the dissolution properties of the polymer matrix [17,18,19] and they can be designed to both increase solubility and enable controlled drug release.

The development of amorphous solid dispersions (ASDs) via hot melt extrusion (HME) has made significant progress and is increasingly recognized as a valuable technique in pharmaceutical formulation [20,21,22]. ASDs improve the solubility and bioavailability of poorly water-soluble drugs by transforming them into an amorphous state, which is generally more soluble than their crystalline counterparts [23]. HME is especially useful in this area because it efficiently disperses active pharmaceutical ingredients (APIs) within a polymer matrix through the application of heat and mechanical shear [24,25]. Its versatility allows for continuous processing, the creation of various dosage forms (like films, tablets, and granules), solvent free process, and enhanced scalability [26,27]. Twin-screw extruders, which are commonly used, provide effective mixing and controlled extrusion conditions [28]. In this study, hot melt extrusion (HME) was evaluated and confirmed as an effective method for producing amorphous solid dispersions (ASDs), which builds on its established use in this field. UV–Vis spectroscopy was employed as a simple and effective alternative for in-line process monitoring, enabling the rapid detection of changes that could affect critical quality attributes, such as drug saturation within the polymer matrix. Piroxicam (PRX) was chosen as the model drug, while Kollidon^®^ VA64 (PVPVA) was used as the matrix, consisting of a vinyl pyrrolidone–vinyl acetate copolymer. This work builds on earlier studies [29,30] and emphasizes the practical application of in-line UV–Vis data to monitor and control the critical quality attributes (CQAs) of extruded products, particularly color parameters such as L*. The results demonstrate the potential of this method to advance process analytical technology (PAT) by providing a reliable, real-time solution for maintaining product quality and enhancing manufacturing processes.

This same spectroscopy tool has been applied in tablet manufacturing to monitor the content uniformity of theophylline tablets in real time [31], adhering to ICH Q2 guidelines for specificity, precision, and accuracy. Tablet density measurements have also been performed using this system, and the CIELAB color space (C* values) showed a strong correlation with density [32]. These findings suggest that color information can effectively measure density, providing a simpler alternative to more complex analyses.

While in-line process analytical technology (PAT) itself is not a new concept, the application of in-line UV–Vis data specifically for monitoring CQAs such as L* (lightness of color) and its integration for real-time process optimization presents new insights into technologies that have not yet been widely implemented, thus contributing to the advancement of the field.

## 2. Materials and Methods

### 2.1. Materials

The active ingredient used in the study was Piroxicam sourced from Medex, Rugby, UK. The polymer carrier employed was Kollidon^®^ VA64 (PVPVA), generously provided by BASF, Ludwigshafen, Germany. The Piroxicam was utilized in its original form as a white powder with crystal structure identified as anhydrous I (AHI), confirmed through powder X-ray diffraction (PXRD) and Raman analysis. Thermal analysis revealed Piroxicam’s experimental glass transition (Tg) at 60 °C, melting point (Tm) ranging between 198 and 202 °C, and a notable weight loss (degradation) with onset observed at 215 °C. According to the biopharmaceutics classification system (BCS), Piroxicam falls under class II, indicating poor water solubility, specifically 23 mg/mL at 22 °C [33,34].

### 2.2. Methods

#### 2.2.1. Upstream Extrusion

Initially, the bulk piroxicam underwent sieving (1.0 mm) to eliminate agglomerates. The powder mixtures were blended using either a tubular mixer (0.5 L), a V-cone mixer (Pharmatech, Birmingham, UK) (1 L), or a mixer (Glatt, Binzen, Germany) (4 L), depending on the batch size required. A gravimetric flex wall single screw hopper (Brabender FlexWall^®^ FW20, Duisburg, Germany) as utilized in conjunction with the load cell controller Congrav^®^ CM-E 1.0 (Brabender Technologie, Duisburg, Germany). This feeding system adjusted the motor speed based on the loss weight to achieve the desired feed rate.

#### 2.2.2. Extrusion

The extrusion process was performed using the Nano16 extruder from Leistritz, Allendale, NJ, USA, featuring twin co-rotating 16 mm screws housed within the barrel. The barrel is equipped with one feeding zone, three heating zones, and a die with a 1 mm diameter. The temperature settings for barrel zones 1 to 3 were 120 °C, 130 °C, and 140 °C, respectively. The powder feed zone was maintained at temperatures between 50 and 60 °C. The die temperature, representing zone 4, is one of the critical process parameters under investigation, with values ranging from 130 to 150 °C.

#### 2.2.3. Downstream Extrusion

Extrudate strands were collected using an air-cooling conveyor belt (Ruhrgetriebe KG, Mülheim an der Ruhr, Germany) to facilitate the solidification of the extrudates. These strands were then continuously transferred to a pelletizer (Accrapak, Warrington, UK), which cut them into 1 mm size pellets. This machine offers controls for adjusting the speed and gap size of the entrance. For analytical characterization, the milling of samples was conducted using a ball-mill MM200 (Retsch, Haan, Germany). Each container was filled with an equal weight of pellets and agitated for 10 min at 10 Hz, resulting in powders with a particle size range of 400–600 µm. For larger quantities of samples, milling was performed using the Quadro^®^ Comill H5 (Quadro-Fitzpatrick, Chesham, UK) at 1500 rpm.

### 2.3. In-Line Analytical Methods

For in-line measurements, the UV–Vis spectrophotometer Inspectro X (ColVisTec, Berlin, Germany) was linked directly to the extruder die. This setup involved two transmission polymer melt probes (TPMP) (ColVisTec, Berlin, Germany). Each UV–Vis probe had a sapphire window, which was self-cleaning due to material flow, along with separable fiber optics for calibration. Illumination was achieved using a broad-band Xenon flash lamp with a fiber length of 5 m. The transmission polymer melt probes (TPMP) were specifically designed for molten polymers, capable of withstanding temperatures up to 325 °C and pressures up to 200 bar. The focal point of each probe is located just outside the durable sapphire lens, ensuring high-precision measurements. These transmittance probes pass light through the molten sample, requiring two probes aligned to face each other. The gap between the probes was measured using steel feeler gauges, with a target range of 0.70–0.80 mm. Before beginning the measurements, a reference signal (dark current) was recorded without a sample, followed by signal optimization according to standard procedures [30]. The probe signal was then adjusted to fall within the optimal range of 40,000–60,000 counts. If necessary, parameters such as the number of flashes and acquisition time were modified to achieve these conditions.

The Equicolor software (ColVisTec, AG, Berlin, Germany) was employed to compute the percentage of transmitted light (transmittance). Data collection spanned from 230 nm to 816 nm with a resolution of 1 nm. The frequency of data collection was 0.5 Hz, and each spectrum was derived from an average of 10 scans. The UV–Vis spectrophotometer probe used had a spot size of 2 mm in diameter, and the measured sample volume typically amounted to 2.5 mm^3^.

L* values were derived from transmittance measurements within the wavelength range of 380–780 nm. The lightness (L*) value, ranging from 0 to 100, serves as an indicator of sample transparency, with values closer to 100 suggesting transparency and values closer to 0 indicating opacity [35]. This parameter is calculated from the spectral tristimulus value Ȳ which is one of the components of the XYZ color space. Also, the transmittance spectrum, relative spectral power and the spectral tristimulus value of the nominally white object (Y_n_) are used in the equations to calculate L*. Detailed equations and their derivations have been documented in [30,32,36] for reference.

### 2.4. Off-Line Analytical Methods

A scanning electron microscope EVO LS 15 (Zeiss, Birmingham, UK) was used to characterize samples appearance. The samples were fixed on the surface of aluminum pin stubs (6 mm height/12.5 mm disk diameter) (Agar scientific, London, UK) using carbon tabs (Agar scientific, London, UK), then coated with gold (15 nm thickness).

A digital microscope Zeiss Smartzoom 5 (Zeiss, Birmingham UK) was used to characterize extruded samples colour and transparency. Strands were fixed in transversal position in a microscope glass slide. The micrographs were taken using objective lens 5× and magnifications 101×, 130× and 1010×. Images were collected using the Smartzoom5 (Zeiss, Birmingham, UK) software with resolutions of 0.22–2.21 μm/pixel.

Differential scanning calorimetry (DSC) experiments were performed using the Polyma 202 (Netzsch, Selb, Germany) DTA, and data therefrom was analyzed using the thermal analysis software Proteus^®^ 80 software (Netzsch, Selb, Germany). Samples were scanned from −50 °C to 230 °C at 10 °C/min under N2 atmosphere (40 mL/min). Plots are available in Appendix SB.

X-ray diffraction (XRD) was also employed to comprehensively characterize the ASD products (Appendix SB supplementary material). The equipment D2 Phaser X-Ray diffractometer (Bruker, Berlin, Germany) was used to analyze physical mixtures (PM) and extrudate samples. Experimental conditions were input by the operation software Diffract.Suite Bruker (Bruker, Berlin, Germany) while data collection was conducted via Diffract.Eva (Bruker, DE) software. Powder samples were manually dispersed into a circular metal sample holder using a glass slide with hand pressure to obtain leveled surface. Samples were subject to measurements from 4.02 to 40.02 2θ angles at step time 0.5 s and step size 0.02–0.006 2θ.

### 2.5. Empirical Models

The JMP Pro 17 software (SAS Institute Inc., Cary, NC, USA), was used to design multivariate experiments, including screening, optimization, and robustness studies, as well as to develop empirical models. The choice of experimental design is closely tied to the selection of the appropriate statistical model.

## 3. Results

### 3.1. Quality Target Product Profile and Critical Evaluation

As per ICH Q8 (R2) [37], pharmaceutical development aims to design a quality product and its manufacturing process to consistently deliver the intended performance. This systematic approach begins with the quality target product profile (QTPP), which is outlined in Table 1 for this study. The intended use, dosage strength, and stability of the quality target product profile (QTPP) were derived from the British National Formulary (BNF). The delivery system, in this case, is not the final product; instead, the target for the amorphous solid dispersion (ASD) is a 16% piroxicam dispersion within the copolymer matrix. The appearance of the product was determined by the die diameter of the extruder, which produced 2.5 mm strands.

**Figure 1:**
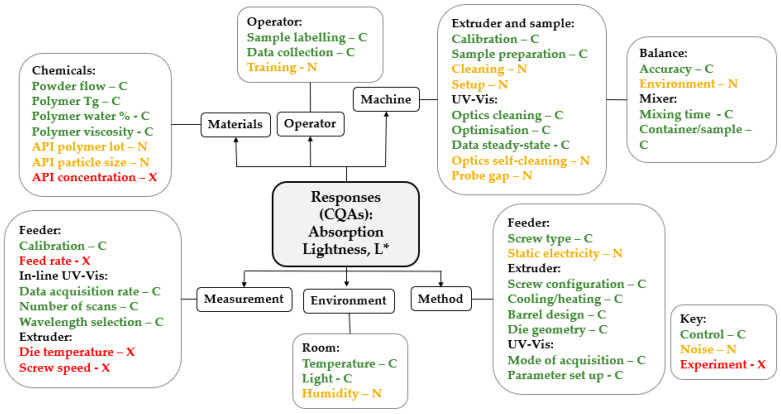
Preliminary risk assessment (Ishikawa diagram) for Piroxicam ASD produced via HME sequential experimental design. The colors illustrate level of risk associated with each source of variability: red = high, amber = medium, green = low.

Given the nature of manufacturing processes and the number of attributes associated with pharmaceutical ingredients, a structured risk assessment (RA) [38] is imperative to identify critical process parameters (CPPs), critical material attributes (CMAs), and critical quality attributes (CQAs). In this study, the identified CPPs include die zone temperature (°C), screw speed (rpm), and solid feed rate (g/min). Additionally, the formulation parameter tested was the API load or concentration (% *w*/*w*). The objective was to comprehend how process and formulation conditions influence ASD product quality, particularly in terms of amorphous content and appearance. Figure 1 shows a preliminary risk assessment of the extrusion process. A cause-and-effect diagram, also known as a fishbone or Ishikawa diagram, serves as an effective mind-mapping tool for identifying and organizing potential variables in development and manufacturing processes. Figure 1 illustrates an example of a fishbone diagram applied to the development and production of piroxicam ASDs. In this context, the terms labeled as CNX are defined as follows: C—factors that are controlled throughout the process; N—factors that cannot be controlled but, when understood, help minimize variability; and X—factors that are potentially critical and require experimentation to assess their influence. For this case, the critical factors identified include temperature, screw speed, feed rate, and concentration. These factors were analyzed in order to determine their relationship with key responses, such as L*.

### 3.2. Sequential Experimental Designs

**Figure 2:**
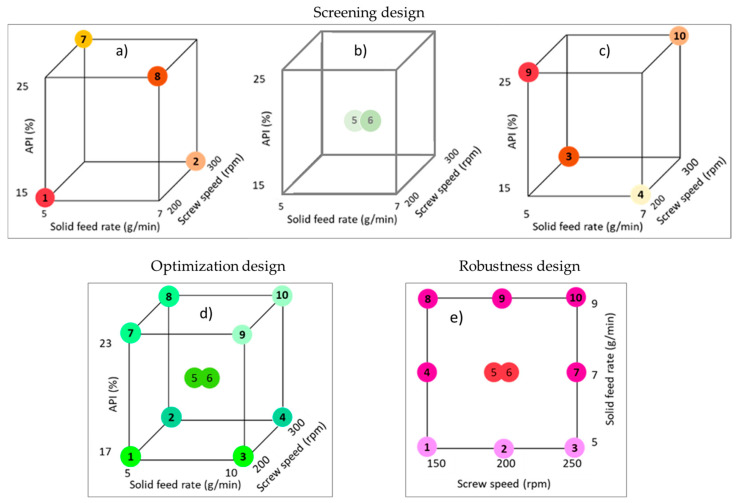
Experimental conditions (runs) for the designs: screening at (**a**) 130 °C, (**b**) 140 °C, (**c**) 150 °C; optimization (**d**) at 140 °C and robustness (**e**) at 140 °C and 16% PRX.

Table 2 outlines the settings of extrusion and formulation parameters employed in the sequential experimental design. The screening experiment entailed four parameters combined using a fractional factorial design, with half of the two-level combinations tested to ascertain main effects. Subsequently, the die temperature remained fixed in the following experimental set, while the other three parameters were organized in a two-level full factorial design. The optimization design comprised eight parameter combinations along with two center points to estimate quadratic contributions, first-order two-factor interactions, and experimental variability. Finally, the robustness experiment involved fixing PRX load and temperature, while investigating screw speed and feed rate settings using a central composite design to ascertain the relationship between these parameters and L*. The designs considered two to four factors at two levels and two midpoints to detect possible interactions and non-linear behavior of the responses, generating a set of runs in each experiment (Figure 2). Appendix SA (supplementary material) provides a summary of the extrusion conditions for all designs including the average values for mechanical energy, specific feed load, and torque for all experiments.

CIELAB color space is a systematic and accurate tool for representing color, where lightness (L*) and the chromaticity coordinates from green to red (a*) and blue to yellow (b*) are defined by the International Commission on Illumination (CIE) [39]. These parameters are calculated from the UV–Vis transmittance spectra collected in real time during the extrusion process. The CQA used as response for the designs proposed (screening, optimization, and robustness) was L* and empirical models were built to understand the relationship between input parameters (concentration, temperature, screw speed, feed rate) and outputs or responses, in this case, L*. This parameter is a valuable response in HME processes for monitoring material uniformity, detecting degradation (as previously reported [30]), and ensuring process consistency. It serves as a quick, non-invasive indicator of issues like incomplete mixing or saturation. When solid material, such as undissolved API, is present, UV–Vis light is scattered, and the L* value decreases immediately. When validated, L* can correlate with critical quality attributes such as saturation, supporting real-time process monitoring. However, it may not capture all quality concerns and should be used alongside other responses for comprehensive quality assurance.

### 3.3. Screening Design of Experiments

A fractional factorial design was employed to screen the impact die temperature (130 °C to 150 °C), screw speed (200 to 300 rpm), feed rate (5 to 7 g/min), and drug concentration loads (15% to 25% PRX) on extrudates CQA, L*. Two center points were included to estimate non-linear correlation and experimental variation.

**Figure 3:**
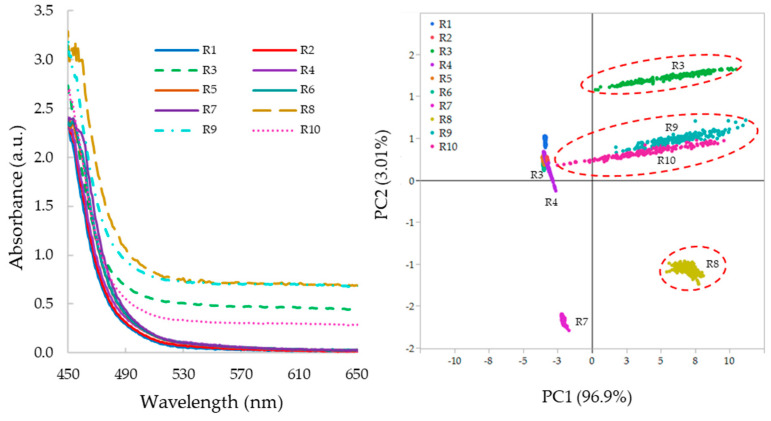
UV–Vis absorbance spectra of samples extruded in the screening design experiment (**left**) and principal component analysis of the 10 samples (**right**).

Figure 3 (left-hand side) illustrates the average absorbance spectra of molten PRX/PVPVA obtained from the in-line UV–Vis measurements. The principal components plot for the absorbance data shows that PC1 accounted for 96.9% of the dataset’s variance, while PC2 represented 3.01% (right-hand side).

Across runs 3, 8, 9, and 10, the spectra exhibited shifts in absorbance intensity throughout the measured wavelength range, resulting in spread over PC1 the positive axis, while runs 1, 2, 4, 5, 6, and 7, PCs clustered along the negative x-axis. This suggests that PC1 captured the variance associated with the presence or absence of light scattering in the samples. In this dataset, the occurrence of light scattering behavior coincided with the presence of bubbles or opacity, likely due to the presence of solid API particles within the samples. These shifts in spectra baseline indicated the presence of light scattering features, as observed in previous studies [26].

**Figure 4:**
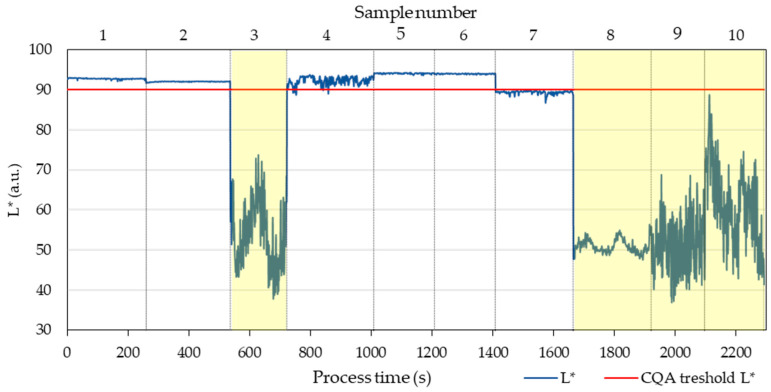
L* values for 10 samples as a function of process time. Samples highlighted in yellow have failed the CQA criterium for transparency and samples 4 and 7 showed low signal to noise ratio, as borderline cases.

Figure 4 illustrates the variation in L* values over process time (in seconds), with vertical lines delineating data from each individual run. Across the dataset, L* values ranged from 36.98 to 94.27. Notably, runs 3, 9, and 10 exhibited high variability in L*, while run 8 demonstrated lower L* values but with reduced variability (ranging from 47.58 to 56.35).

**Figure 5:**
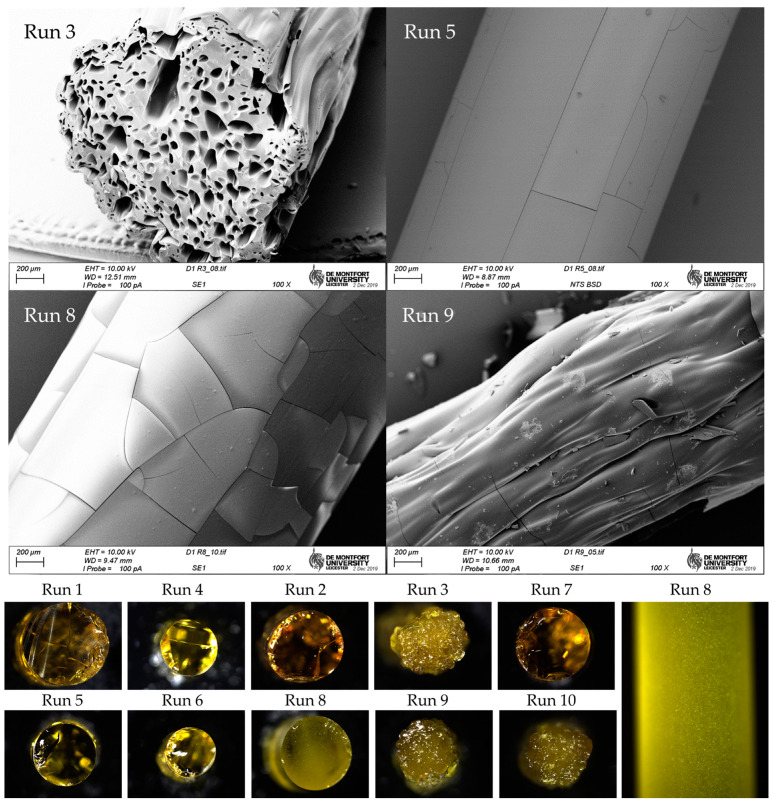
**Top**: scanning electron microscopy (SEM) images of samples 3, 5, 8, and 9 from DoE1. **Bottom**: optical microscopy images of all samples from DoE1 (cross-sectional view) and a top view of sample 8, highlighting its opaque appearance.

Scanning electron microscopy (SEM) images (Figure 5 top) revealed bubbles within the extruded strand for run 3 and burst marks from bubbles on the surface for run 9. The microscopy images highlighted differences in surface attributes, with run 8 strands featuring minor round relief marks, while run 5 strands exhibited a smoother surface. Light microscopy images (Figure 5 bottom) indicated that despite having a smooth surface devoid of bubbles, run 8 still exhibited an opaque appearance. This opacity is attributed to the presence of solid particles, possibly resulting from saturation or precipitation of the active pharmaceutical ingredient (API).

The statistical analysis outputs for the L* model based on process and API % parameters are summarized in Appendix SC. The R^2^ adjusted value (adjusted to take into account only the terms used in the model) indicated that the model explained 95.09% of the observed variability. The sorted parameter estimates show model terms based on t-Ratio values, indicating the factors that had the most significant impact on L* values.

The interaction between API % and screw speed emerged as the most influential factor, with a t-Ratio of 110.91, followed by the quadratic term for API%. This quadratic term highlighted the scattering issues predominantly observed in runs with 25% PRX. Furthermore, temperature, API %, and the interaction between API % and feed rate exhibited similar magnitude effects on L* values, with t-Ratios of −85.91, −84.86, and −84.63, respectively. Although the independent factors of screw speed and feed rate were statistically significant, it was their interaction with API % that primarily explained the variation in L*.

**Figure 6:**
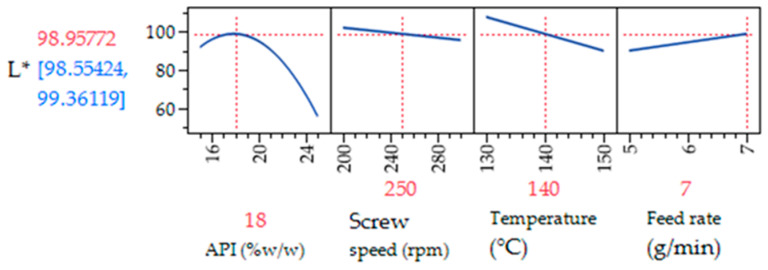
Prediction profiler for the screening design of experiment runs, showing how the 4 factors impact the CQA L*. The vertical red dotted lines represent the values of the four parameters that yield the optimal value for L*.

The prediction profiler plots depict the trends in the statistical model. Figure 6 illustrates the maximum L* value achieved for PRX concentration of 18%, screw speed of 250 rpm, temperature of 140 °C, and feed rate of 7 g/min. The correlation between drug load and L* exhibits a quadratic behavior, being positive until reaching a peak around 18%, and then becoming negative for concentrations exceeding 20%. The predicted values presented in the model, although derived from fitting to the screening design of experiments data, have limitations. Specifically, L* values exceeding 100 do not accurately represent the lightness scale but rather constitute an extrapolation of the predicted trend.

Additionally, the model indicated that higher die temperatures result in decreased L* values. This trend aligns with experimental observations, as most samples processed at 150 °C exhibited lower L* values attributed to the presence of scattering features caused by bubbles. The majority of unsuccessful samples were associated with the highest tested drug load (25% PRX). Samples tended to exhibit an opaque appearance when subjected to drug loads exceeding 20% PRX, with the exception of sample run 7.

### 3.4. Optimization Design of Experiments

The second design of experiments focused on assessing the impact of screw speed, feed rate, and PRX% on extrudates CQAs. The PRX% was deliberately set to range between 17% and 23% to evaluate the effects of process parameters on the solubility of the PRX around the saturation limit, which was determined to be 20% in the screening experiment. Screw speed was varied from 200 to 300 rpm, while the feed rate ranged from 5 to 10 g/min. The wider range of feed rates included higher throughput compared to the screening experiments, aiming to examine the effects of residence time on the L* (Design depicted in Figure 2d).

The die set temperature was maintained at 140 °C, with a maximum allowable variation of ±5 °C. The decision to fix the temperature at 140 °C was based on two primary considerations. Firstly, this temperature represents the lowest feasible value for processing pure Kollidon^®^ VA64 with the equipment employed. Secondly, this temperature was chosen to prevent API decomposition, as reported in previous studies [29]. By maintaining the temperature within this range, the experiment aims to ensure the integrity of the API and the stability of the formulation throughout the processing.

**Figure 7:**
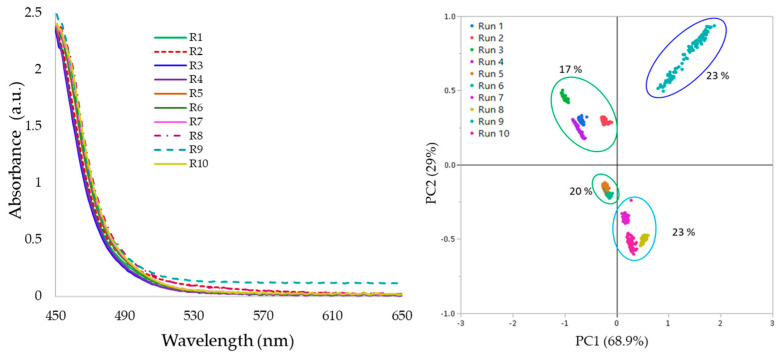
UV–Vis absorbance spectra of samples extruded in the optimization design of experiments (**left**) and principal component analysis of the 10 samples (**right**).

Figure 7 (left-hand side) depicts the average UV–Vis absorbance spectra. Process conditions in runs 2, 8, and 9 resulted in spectra with shifted absorbance values. Spectra collected for run 9 shifted throughout the wavelength monitored (230–780 nm), while runs 2 and 8 spectra shifted only in the region 500–600 nm. The principal component analysis plot highlighted differences in the absorbance data within 460–700 nm. The first principal component represented 68.9% of the dataset variability, while the second described 29%. The first and second principal component scores plot separated run 9 from the remainder of the dataset (right-hand side).

The different scores distinguished the full spectral (230–780 nm) shift connected to the extrudate opacity. Across the PC2 (y-axis), samples clustered in three groups according to PRX%, and this pattern indicated that PRX% influenced spectral features other than those attributable to the baseline shift alone.

**Figure 8:**
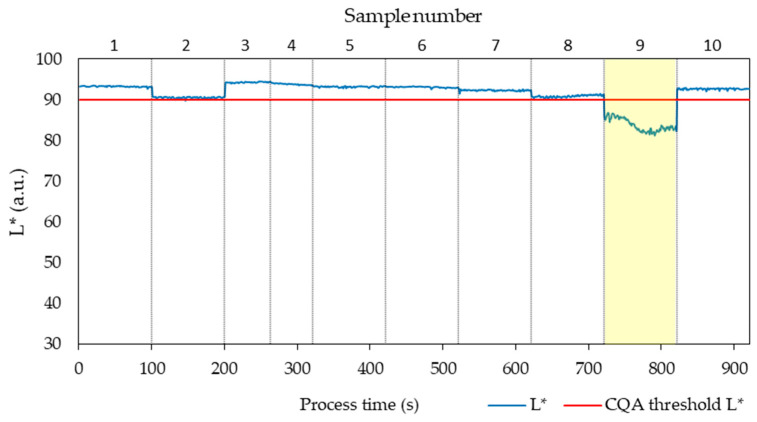
L* values for all 10 samples as a function of process time. Sample highlighted in yellow has failed the CQA criterium for transparency.

Overall, the L* values obtained 81 to 95, so higher and with narrower range compared to those measured in the screening experiment. Only the conditions of run 9 produced samples with light scattering features, resulting in an average lightness value of 83.91 (Figure 8).

L* model statistics outputs are summarized in Appendix SC. According to the R^2^ adjusted value, this model explained 88.20% of the dataset variability. Moreover, the F Ratio = 983.48 suggested that the model was statistically significant with a high signal-to-noise ratio. The t-Ratio column highlighted model terms with the highest impact in L* value. For example, the interaction between screw speed and feed rate had the largest positive effect (t-Ratio = 44.27), followed by API % (t-Ratio = −42.66), and interactions between API% and feed rate (t-Ratio = 35.63), and screw speed (t-Ratio = 30.68).

**Figure 9:**
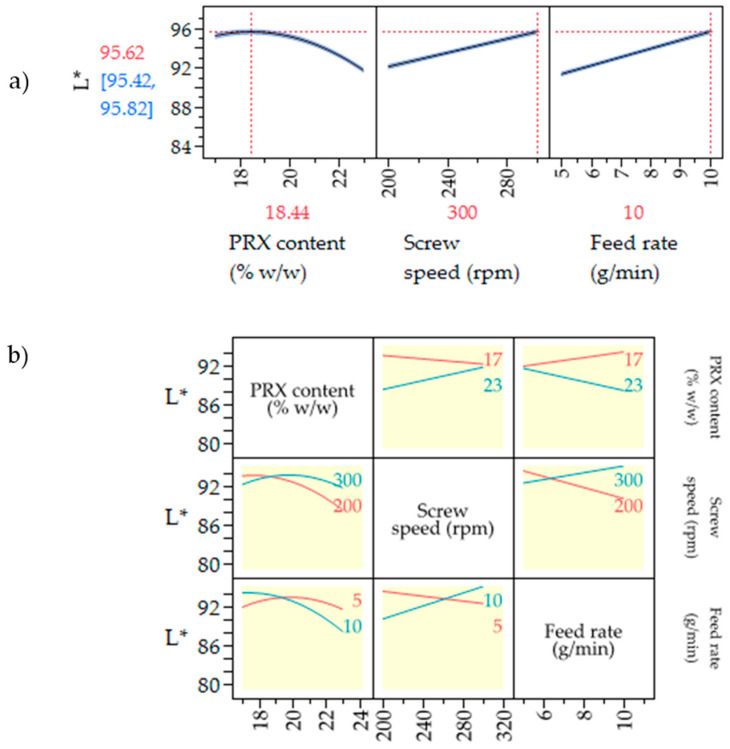
(**a**) Prediction profiler for L* values vs. optimization design of experiments parameters. The vertical red dotted lines represent the values of the three parameters that yield the optimal value for L*. (**b**) Interaction profiles between the 3 factors investigated for the CQA L*. API % interacts with screw speed and feed rate (highlighted in yellow). The red and blue colors represent the trends between L* and the other parameters (i.e., PRX content, screw speed, and feed rate) for high (blue) and low (red) levels to evaluate the interactions between two parameters.

Figure 9a depicts prediction profilers for L* values. According to the model predictions, L* values have a curvature behavior as a function of PRX%, varying from 92.93 to 89.84 when the PRX% increased by 6%. The prediction profile shows that L* changed across PRX% because of interactions between the drug load and process parameters. For example, at 17% PRX, the screw speed negatively impacts L*, while at 23% PRX, this relationship becomes positive.

Process parameter effects were more prominent at higher rather than lower drug load because the formulations tested were around the drug limit of saturation (~20%). At 17% PRX, the drug was fully dissolved independently from the process screw speed and feed rate. In contrast, processing a 23% PRX formulation resulted in extruded samples with different attributes, transparent or cloudy, depending on the combination of screw speed or feed rate. Overall, the model demonstrated that interactions between screw speed, feed rate and PRX% impacted L* (Figure 9b).

### 3.5. Robustness Design of Experiments

The robustness runs (design depicted in Figure 2e) evaluated the robustness of the UV–Vis signal within a range of process conditions. In this design, the concentration and temperature were held constant at 16% and 140 °C, respectively. However, the feed rate and screw speed were varied within the ranges of 5–9 g/min and 150–250 rpm, respectively. The formulation drug load was deliberately set to 16% PRX to ensure it was far from the saturation boundary (>20% PRX) to minimize the risk of failure.

**Figure 10:**
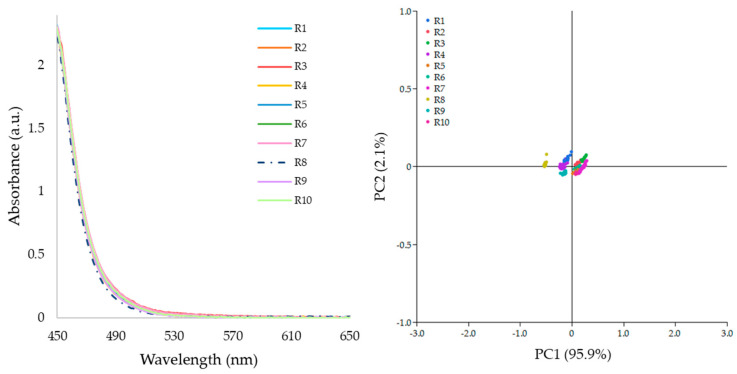
UV–Vis absorbance spectra of samples extruded in the robustness design experiment (**left**) and principal component analysis (**right**).

Figure 10 shows that absorbance spectra are almost identical independent of the extrusion process conditions and have only minor differences around 480–580 nm. The absence of spectra baseline shift indicated that all runs extruded transparent strands without bubbles and opacity. The principal components analysis applied to spectra in the range 460 to 700 nm resulted in a first principal component representing 97.5% of the dataset variability, while the second described 1.99%. The small distance between data points demonstrated that spectra do not show trends related to process conditions. In other words, the samples produced using the robustness design of experiments have very similar spectral features.

**Figure 11:**
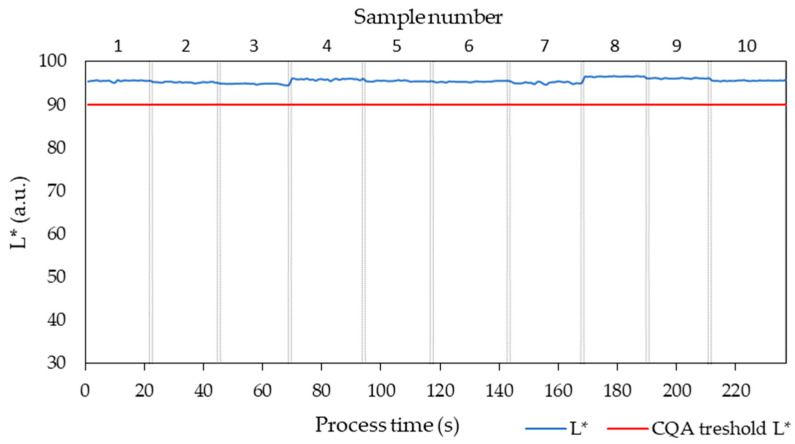
L* values for all 10 samples as a function of process time. All samples passed the CQA criterium for transparency.

In the robustness design experiments dataset, the L* values ranged from 94.72 to 96.52, so all samples passed the CQA criteria of L* > 90 (Figure 11). This information is relevant to further research stages where the L* signal could be used to monitor the quality of the extrusion products. The statistical analysis for L* model is summarized in Appendix SC. The value of F Ratio (signal to noise ratio) is 517.82. The R^2^ adjusted values indicated the model for L* described 91.63% the dataset variability. Thus, the statistical analysis confirmed that the model fitted well the dataset. The t-Ratio estimated that L* was affected by screw speed and feed rate.

**Figure 12:**
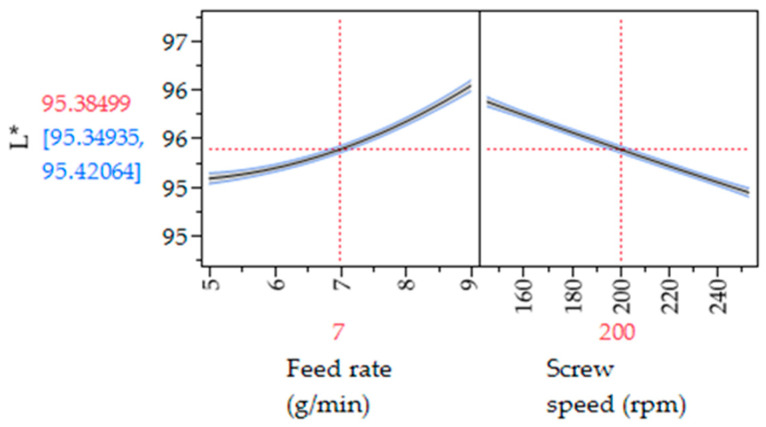
Prediction profiler for L* values vs. robustness design of experiments parameters. The vertical red dotted lines represent the values of the two parameters that yield the optimal value for L*.

**Figure 13:**
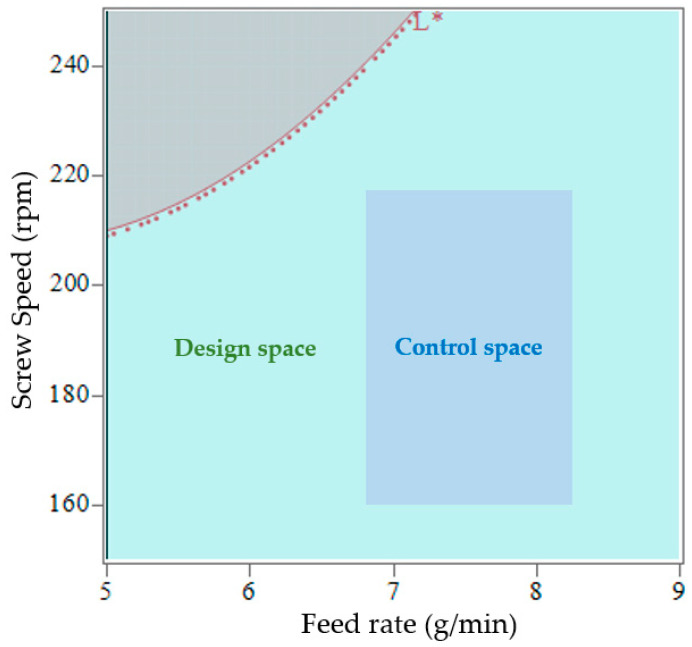
Design space (green area) for the robustness study for an ASD containing 16% of PRX and extrusion temperature of 140 °C. The blue area represents a potential control space for efficient manufacturing, while the grey area should be avoided, as products in this region are likely to fail to meet quality target specifications. The red dotted line marks the L* contour, delineating the boundary between pass and fail.

Figure 12 presents the prediction profiler for L* values from the robustness design of experiments. The predicted mean L* varied slightly, from 95.42 to 95.34. The lack of influence of process parameters on L* values was due to the fixed PRX% used in this experiment. In a 16% PRX formulation, the API could be fully dissolved into the polymer, so the product is expected to be transparent independently of process conditions. If the threshold for L* is 90, the design space for the robustness experiments is the entire range for screw speed (150–250 rpm) and feed rate (5–9 g/min). All samples have L* values greater than 94. This study defines the extrusion design space using the in-line UV–Vis lightness quality at-tribute (L*), illustrated as the light green area in Figure 13. In practice, a control space can be established within the design space, consisting of operating settings monitored and managed during routine manufacturing. The limits are determined by the ease with which the process can be controlled, taking into account the equipment and strategies employed in the manufacturing process. The gray-shaded area represents regions to avoid, as products within this range will not meet the quality target profile. The red-dotted contour line delineates the boundary between failure and compliance with the quality target.

## 4. Discussion

This study showed how critical process parameters such as temperature, screw speed, and feed rate impacted the solubilization of PRX in PVPVA within the drug load range (15 to 25%) when processed by hot melt extrusion.

The screening experiments demonstrated how PRX % and temperature affected the extruded ASDs L* value. The combination of high temperature (150 °C) and high screw speed (300 rpm) resulted in bubbles inside the extruded product arising from the drop in the molten polymer viscosity, hence, results in a higher frequency of bubbles in experiments with a drug load above 20% PRX.

The appearance of the extruded products indicated the API level of solubilization in the polymeric carrier. Taylor et al. have also related solid dispersion transparent appearance to the amorphous state of the active ingredient in ASDs or ‘solid solution’ [40]. The non-transparent appearance of the extruded samples was attributable to the presence of the drug in the crystalline state dispersed in the polymer carrier.

The in-line UV–Vis spectra detected the effects of formulation and process parameters in the PRX solubilization in the PVPVA matrix. Processing a formulation with 25% PRX at 130 °C temperature and 200 rpm screw speed caused a full-spectrum baseline shift and low lightness values. Previous work [29] reported similar shifts on the baseline being attributed to saturation of the API.

Although higher process temperatures and screw speeds produced samples without residual crystallinity (confirmed by XRD), they introduced other failure modes, such as bubble generation. While bubbles might not pose a fundamental issue, their presence scatters light, potentially leading to false conclusions about saturation due the sample being opaque. The difficulty in dissolving the PRX at concentrations higher than 20% PRX at different processing conditions indicated that the formulation drug load should be kept below 20% PRX, and the process temperature as low as possible, whilst retaining the torque below 18 Nm (for the extruder used in this work). It is important to bear in mind that pure PVPVA cannot be processed at temperatures below 140 °C when using this extruder in view of its high viscosity below this temperature.

In the optimization experiment, a decision was made to maintain the temperature at a constant value (140 °C), and to narrow the range of the drug load, but using a maximum value (23%) that would be just above the saturation point (20–21%). The results showed that feed rate and screw speed settings affected the optical properties of samples with 23% PRX; however, these process parameters had opposite effects in extruded product transparency. The statistical model indicated that the solubilization of PRX 23% could improve by combining feed rate and screw speed of 7 g/min/200 rpm or 10 g/min/300 rpm. For example, samples with the same feed rate produced opaque/transparent dispersions at different screw speed settings. A high screw speed selection generated more transparent products arising from the additional mechanical energy provided to disperse the drug in the carrier. Screw speed affects residence time distribution, but this is a minor effect if compared with feed rate, as reported in [27]. Since the feed rate can decrease the molten mixture residence time, consequently, it reduces the heat transfer between molten material and the extruder barrel required to soften the polymeric carrier and solubilize the drug. To optimize the extrusion process, it is important to seek and find the balance between screw speed and feed rate, since screw speed contributes to increased temperature in view of the shear mechanical energy input and feed rate controls molten material residence time [22,24]. The feed rate increases the process throughput and productivity, but a higher barrel-specific feed load could result in higher torque and powder feed overflow, both considered extrusion failure modes [25].

A drug load of 16% PRX was selected to operate the process far from the saturation limit (20–21%). The process temperature was fixed at 140 °C which yielded amorphous dispersion with desirable mechanical stability. The screw speed upper limit was set at 250 rpm to prevent PRX color changes associated with excessive mechanical shear. The robustness experiments demonstrated that the extrusion conditions provided by the optimization experiment (PRX < 20%, 150–250 rpm, 5–9 g/min) produced an amorphous solid dispersion with desired critical attributes of a low level of variability.

Similarly, research by Wesholowski et al. [41,42] explored the application of in-line UV/Vis spectroscopy as a PAT tool for HME. Their work emphasized the potential of this technique for real-time monitoring of solid dispersion preparations, underscoring its utility in ensuring product quality and process efficiency. Additionally, a study by Netchacovitch et al. [43] validated an in-line Raman spectroscopic method for continuous API quantification during HME processes. While focusing on Raman spectroscopy, their work contributes to the broader field of in-line spectroscopic techniques for real-time monitoring and control in pharmaceutical manufacturing. A recent review published by Zhang et al. [44] highlights the essential steps in developing a PAT strategy for hot melt extrusion (HME), and emphasizes the role of chemometrics in processing and interpreting complex data. It presents three key process analyzers: near-infrared (NIR) spectroscopy, Raman spectroscopy, and ultraviolet/visible (UV–Vis) spectroscopy for obtaining critical process data. While significant progress has been made in applying PAT strategies to pharmaceutical HME, challenges such as algorithm limitations, regulatory compliance, and a lack of suitable process analyzers remain.

This work contributes to the growing body of research demonstrating the efficacy of in-line UV–Vis spectroscopy combined with DoE methodologies in HME processes. By focusing on L* as a critical quality attribute, we offer a practical approach to real-time monitoring and control, supporting the production of high-quality ASDs and advancing the field of pharmaceutical manufacturing.

## 5. Conclusions

This study successfully applied a sequential design of experiments to develop models for predicting the dependent variable, lightness (L*). The in-line UV–Vis measurements successfully monitored the critical quality attribute of extrusion products (L*), leading to process optimization. Furthermore, the in-line system characterized samples without any sample preparation apart from the manufacturing process itself. Measuring L* is particularly important for process monitoring and control in the production of amorphous solid dispersions (ASDs), where maintaining consistency is crucial for product performance and quality. L* provides a simple, real-time indicator of the physical appearance of the ASD, which can be directly linked to the uniformity of the dispersion and stability of the process. Small variations in L* can signal changes in key processing conditions—such as concentration of API, temperature, mixing, or phase separation, that could affect the amorphous state or the homogeneity of the dispersion. By tracking L* continuously during production, manufacturers can detect these issues early, preventing deviations from the desired product characteristics.

As a straightforward and easy-to-measure output, L* serves as an effective tool for monitoring the quality of the dispersion without the need for complex and time-consuming analyses. Moreover, L* can potentially be used for process control, enabling automated adjustments to parameters like processing speed, temperature, ensuring that the ASD remains within desired specifications (QTPP). This approach reduces the risk of product variability, minimizes waste, and ensures that the final amorphous solid dispersion consistently meets performance standards. In this way, measuring L* not only simplifies the monitoring process but also supports tighter control over the production of high-quality ASDs, enhancing both efficiency and reliability in manufacturing. In conclusion, this study highlights the practical application of in-line UV–Vis data for monitoring and controlling CQAs of extruded products, particularly L*. The findings show the potential of this approach to advance the field of process analytical technology by offering a robust, real-time solution for ensuring product quality and optimizing manufacturing processes.

## Figures and Tables

**Table 1:** Quality target product profile (QTPP) for the ASD intermediate product.

Quality Attribute	Quality Target Product Profile
Intended use (proposed indication)	Relief of rheumatoid arthritis and osteoarthritis symptoms
Active pharmaceutical ingredient	Piroxicam
Dosage strength	20 mg
Delivery system	16 ± 3% piroxicam amorphous solid dispersion in vinylpyrrolidone–vinyl acetate copolymer.
Appearance	Transparent strands of 2.5 mm diameter
Stability	Amorphous piroxicam content is stable for at least 18 months

**Table 2:** Critical process parameters’ ranges for the sequential experimental designs: screening, optimization, and robustness.

Experiment Objective		Screening	Optimization	Robustness
Experiment design		Fractional factorial	Full factorial	Central composite
N^0^ of experiments		10	10	10
Formulation parameter	PRX load (%)	15–25	17–23	16
Critical process parameters	Die temperature (°C)	130–150	140	140
	Screw speed (rpm)	200–300	200–300	150–250
	Solid feed rate (g/min)	5–7	5–10	5–9

## Data Availability

The data presented in this study are available on request.

## Supplementary Materials

The following supporting information can be downloaded at: https://www.mdpi.com/article/10.3390/pharmaceutics17020151/s1. Appendix SA—experimental conditions for the three sequential experimental designs. Appendix SB—DSC and XRD spectra of samples from screening, optimization and robustness designs. Appendix SC—statistical information for the sequential experimental designs.
