# Supplementary material for: Enhancing Process Control and Quality in Amorphous Solid Dispersions Using In-Line UV–Vis Monitoring of L* as a Real-Time Response
Source: Pharmaceutics. 2025 Jan 23;17(2):151. doi: 10.3390/pharmaceutics17020151 (PMC11859203; doi:10.3390/pharmaceutics17020151)
Supplement: Supplementary file 1 [file pharmaceutics-17-00151-s001.zip › pharmaceutics-3403488-supplementary.pdf]

Enhancing Process Control and Quality in Amorphous Solid Dispersions Using In-Line UV–Vis Monitoring of L* as a Real-Time Response

Mariana Bezerra, Juan Almeida, Matheus de Castro, Martin Grootveld and Walkiria Schlindwein

Supplementary materials

Appendix SA – Design of experiments run conditions (Screening, optimisation, robustness)

Appendix SB – Off-line characterisation (DSC, FTIR)

Appendix SC – Statistical analysis summary (Screening, optimisation, robustness)

Appendix SA

A-1 Screening design experimental runs

Fractional factorial experimental design runs for 4 factors at 2 levels.

Run code	PRX conc. (%w/w)	Screw speed (rpm)	Solid Feed rate (g/min)	Die Temp (°C)	Specific mechanical energy (kW.hkg^{-1})	Specific feed load (-)	Torque (Nm)
1	15	200	5	130	1.14	0.0203	16.49
2	15	300	7	130	1.11	0.0190	15.00
3	15	300	5	150	1.54	0.0136	14.87
4	15	200	7	150	0.87	0.0285	17.70
5	20	250	6	140	1.06	0.0195	14.81
6	20	250	6	140	1.09	0.0195	15.22
7	25	300	5	130	1.36	0.0136	13.19
8	25	200	7	130	0.76	0.0285	15.35
9	25	200	5	150	1.01	0.0203	14.66
10	25	300	7	150	0.96	0.0190	12.95

A-2 Optimisation design experimental runs

Optimisation experimental design runs for 3 factors at 2 levels.

Run code	PRX conc. (%w/w)	Screw speed (rpm)	Solid Feed rate (g/min)	Die Temp (°C)	Specific mechanical energy (kW.hkg^{-1})	Specific feed load (-)	Torque (Nm)
1	17	200	5	140	1.22	0.0203	17.62
2	17	300	5	140	1.55	0.0136	14.98
3	17	200	10	140	0.65	0.0407	18.93
4	17	300	10	140	0.79	0.0271	15.22
5	20	250	7.5	140	0.86	0.0244	14.89
6	20	250	7.5	140	0.86	0.0244	14.93
7	23	200	5	140	1.02	0.0203	14.82
8	23	300	5	140	1.37	0.0136	13.21
9	23	200	10	140	0.58	0.0407	16.91
10	23	300	10	140	0.68	0.0271	13.15

A-3 Robustness design of experimental runs

Robustness experimental design runs for 2 factors at 2 levels.

Run code	PRX conc. (%w/w)	Screw speed (rpm)	Solid Feed rate (g/min)	Die temp (°C)	Specific mechanical energy (kW.hkg^{-1})	Specific feed load (-)	Torque (Nm)
1	16	150	5	140	0.91	0.0271	17.63
2	16	200	5	140	1.11	0.0203	16.16
3	16	250	5	140	1.31	0.0163	15.18
4	16	150	7	140	0.72	0.0380	19.51
5	16	200	7	140	0.84	0.0285	17.08
6	16	200	7	140	0.84	0.0285	16.98
7	16	250	7	140	0.93	0.0228	15.14
8	16	150	9	140	0.58	0.0488	20.06
9	16	200	9	140	0.67	0.0366	17.53
10	16	250	9	140	0.74	0.0293	15.45

Appendix SB Off-line characterisation

Differential scanning calorimetry (DSC) and X-Ray diffraction (XRD) results

DSC and XRD data have been used to confirm API amorphousness in the solid dispersions. The absence of a melting peak in the DSC endotherms corroborated the hypothesis of the API amorphous state. ASD presented a diffuse PXRD pattern which is characteristic of amorphous materials. First heating scan endotherms of PRX, PVPVA, their physical mixture (PM) and extruded samples produced during the screening design and optimisation design experiments are presented. Polymer, physical mixtures and extruded samples presented a broad endothermic peak from 60 °C to 160 °C, attributed to the release of non-bounded water from the polymeric matrix. No endothermic peak was observed at the expected PRX melting temperature.

B-1 Screening design experiments

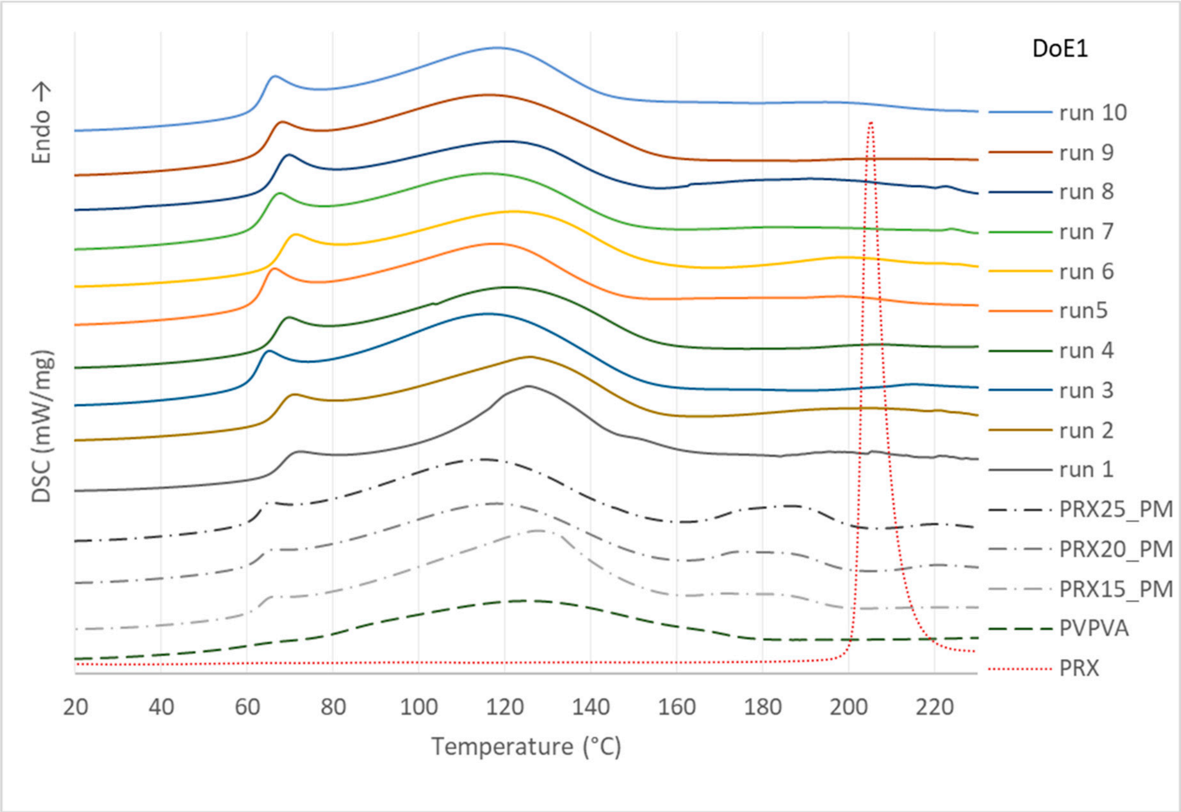

DSC endotherms of reference materials, physical mixtures, and runs 1-10 screening design of experiments extrudates

In the screening design dataset, run 8, which contained 25 % PRX and extruded at 130 °C, showed small intensity peaks at 8.9, 11.6 and 17.54 2 theta values. This pattern indicated the presence of crystalline particles in this sample.

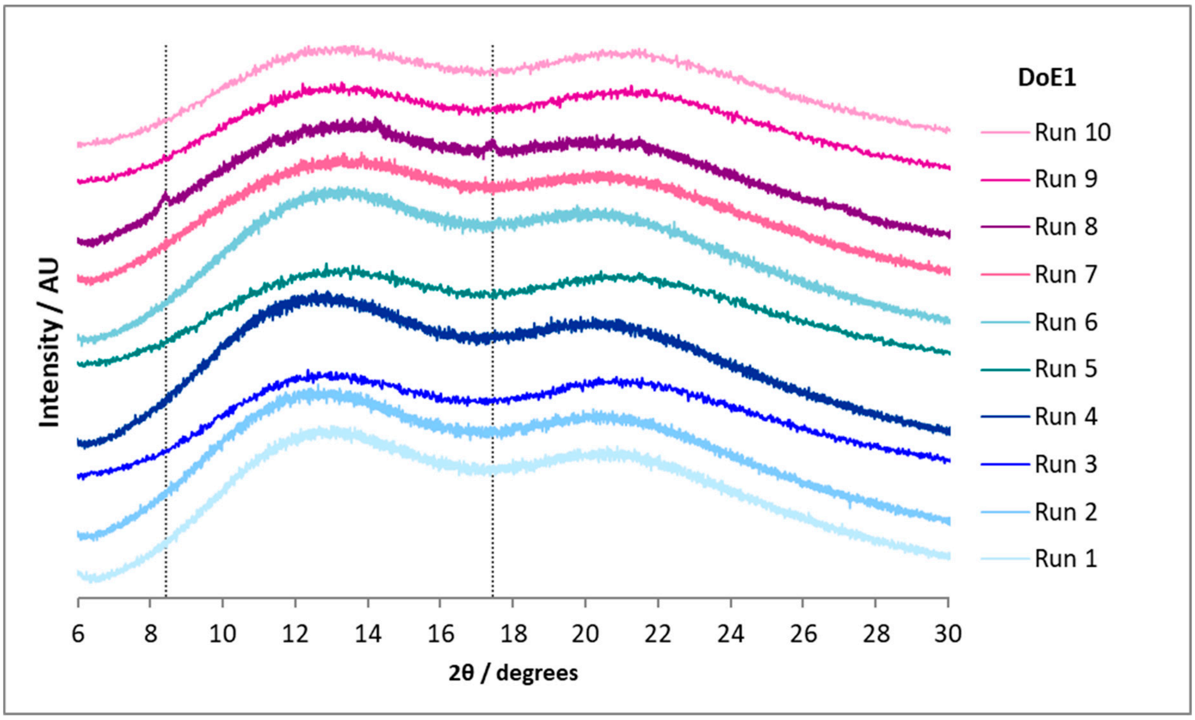

Diffractogram of PRX solid dispersion samples from screening design of experiments

B-2 Optimisation design experiments

Melting endotherms are not detected because of the distance between molecules in glass solid solutions. There is no rigid pattern of molecular arrangement, and no specific temperature loosens the interaction between molecules.

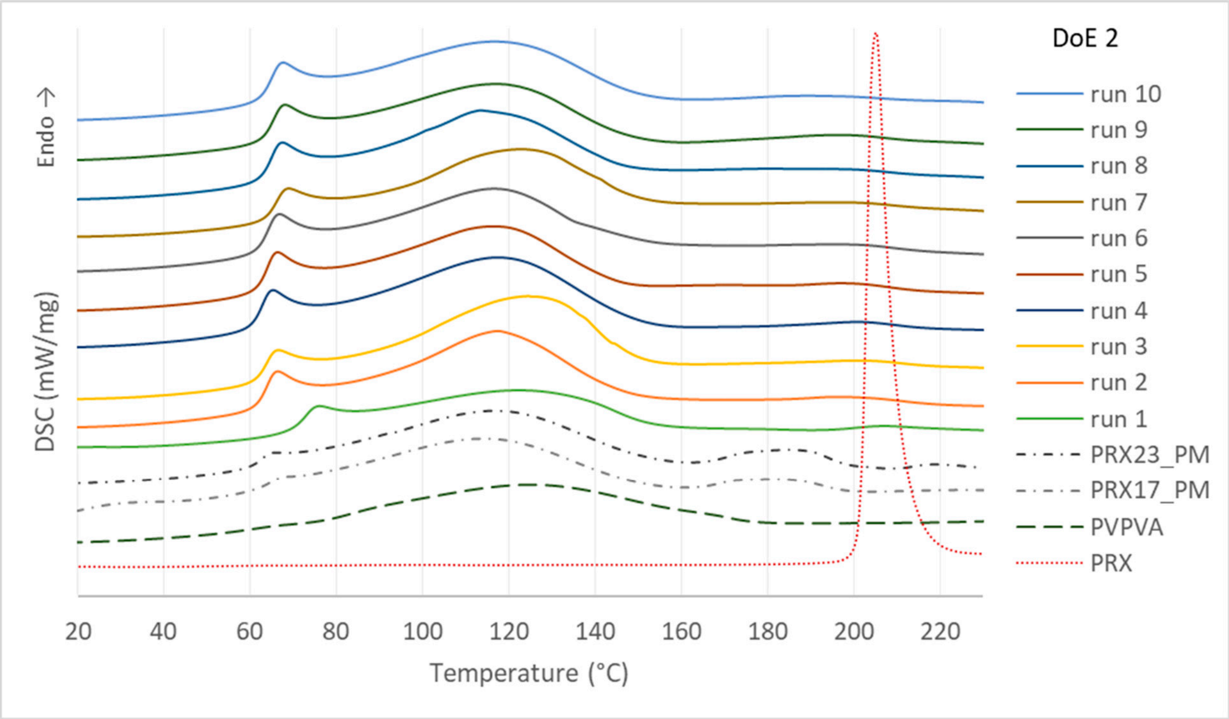

DSC endotherms of reference materials, physical mixtures and optimisation design of experiments extrudates.

In optimisation design XRD data set, all samples presented a hallo pattern interpreted as evidence of an amorphous state.

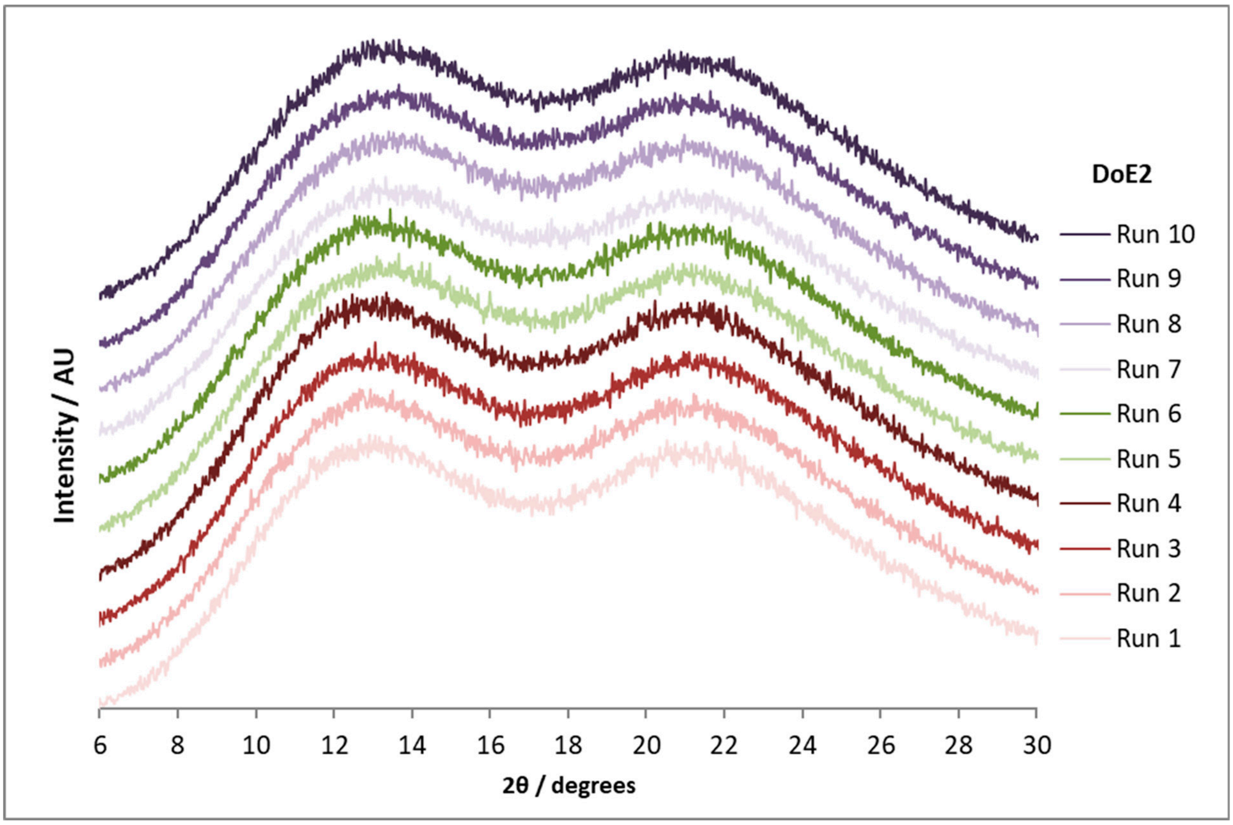

Diffractogram of PRX solid dispersion samples from optimisation design of experiments.

B-3 Robustness design of experiments

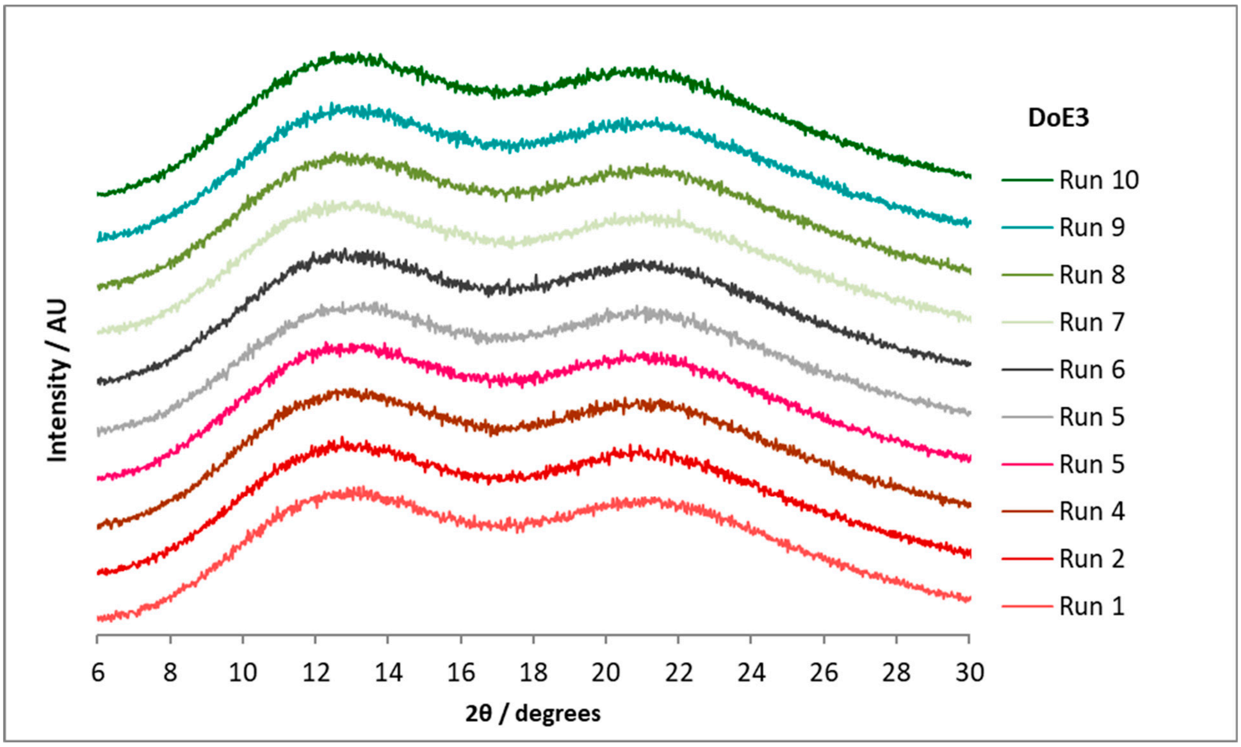

Diffractogram of PRX solid dispersion samples from robustness design of experiments

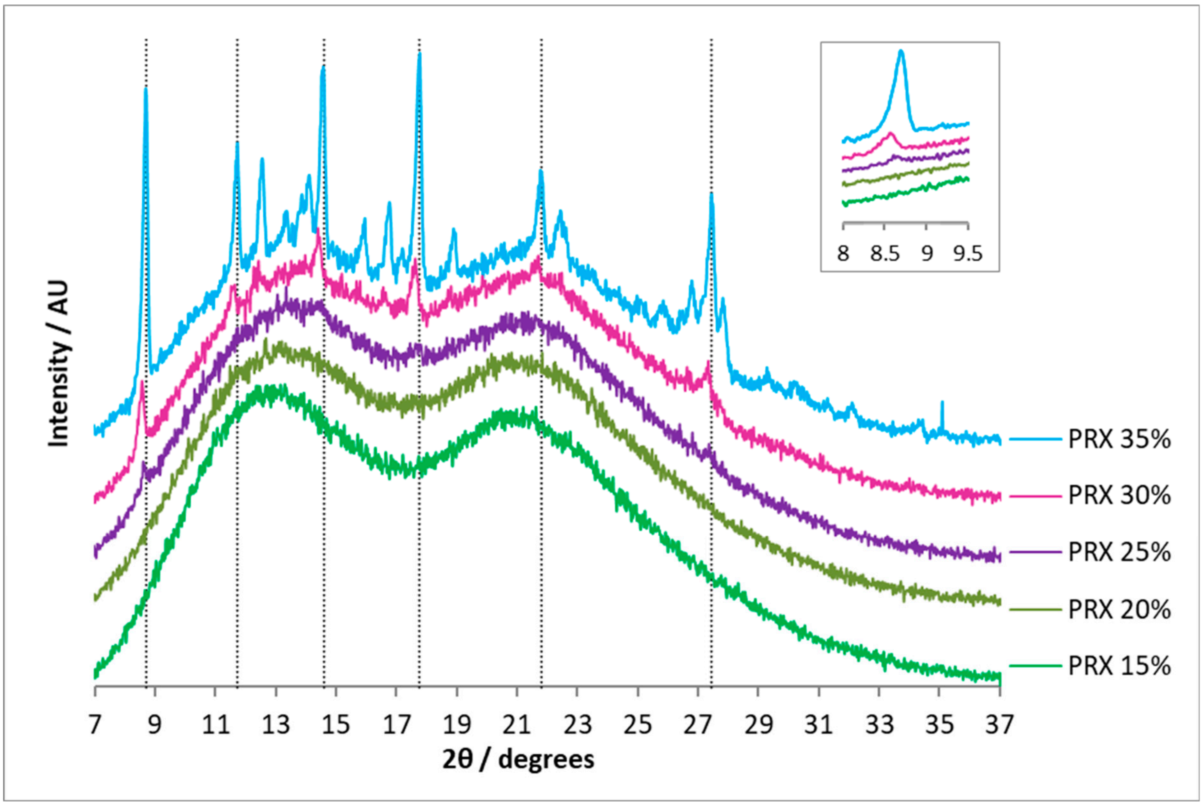

Diffractogram of PRX PVPVA samples extruded at 140 °C, 7 g/min and 200 rpm. The expanded range show that extruded samples don't show diffraction peaks when PRX % is below 25%.

Appendix SC

C-1 Screening design statistical analysis

Summary of statistical analysis of L* model based on screening design of experimental data.

Summary of Fit

RSquare	0.951096
RSquare Adj	0.950924
Root Mean Square Error	4.18555
Mean of Response	78.7335
Observations (or Sum Wgts)	2292

Analysis of Variance

Source	DF	Sum of Squares	Mean Square	F Ratio
Model	8	777836.58	97229.6	5550.004
Error	2283	39995.49	17.5	Prob > F
C. Total	2291	817832.06		<.0001*

Sorted Parameter Estimates

Term	Estimate	Std Error	t Ratio	Prob>|t|
(API (%w/w)-19.7295)*(Screw speed (rpm)-248.604)	0.0433926	0.000391	110.91	<.0001*
(API (%w/w)-19.7295)*(API (%w/w)-19.7295)	-0.841974	0.00924	-91.12	<.0001*
Temperature (°C)	-0.838579	0.009761	-85.91	<.0001*
API (%w/w)	-1.685439	0.019861	-84.86	<.0001*
(API (%w/w)-19.7295)*(Feed rate (g/min)-6.06021)	-1.655499	0.019562	-84.63	<.0001*
Feed rate (g/min)	1.5323248	0.097613	15.70	<.0001*
(API (%w/w)-19.7295)*(Temperature (°C)-139.101)	0.0263373	0.001956	13.46	<.0001*
Screw speed (rpm)	0.01057	0.001952	5.41	<.0001*

C-2 Optimisation design statistical analysis

Summary of statistical analysis of L* model based on optimisation design of experiments data.

Summary of Fit

RSquare	0.88291
RSquare Adj	0.882012
Root Mean Square Error	1.021024
Mean of Response	91.52144
Observations (or Sum Wgts)	921

Analysis of Variance

Source	DF	Sum of Squares	Mean Square	F Ratio
Model	7	7176.9105	1025.27	983.4842
Error	913	951.7939	1.04	Prob > F
C. Total	920	8128.7044		<.0001*

Sorted Parameter Estimates

Term	Estimate	Std Error	t Ratio	Prob>|t|
(Screw speed (rpm)-249.729)*(Feed rate (g/min)-7.28556)	0.0136546	0.000308	44.27	<.0001*
PRX content (% w/w)	-0.572713	0.013425	-42.66	<.0001*
(PRX content (% w/w)-20.2573)*(Feed rate (g/min)-7.28556)	-0.185	0.005192	-35.63	<.0001*
(PRX content (% w/w)-20.2573)*(Screw speed (rpm)-249.729)	0.0078859	0.000257	30.68	<.0001*
(PRX content (% w/w)-20.2573)*(PRX content (% w/w)-20.2573)	-0.187211	0.009114	-20.54	<.0001*
Screw speed (rpm)	0.0125518	0.000761	16.49	<.0001*
Feed rate (g/min)	-0.167173	0.015444	-10.82	<.0001*

C-3 Robustness design statistical analysis

Summary of L* model fit based on robustness experimental data.

Summary of Fit

RSquare	0.918089
RSquare Adj	0.916316
Root Mean Square Error	0.148873
Mean of Response	95.48505
Observations (or Sum Wgts)	237

Analysis of Variance

Source	DF	Sum of Squares	Mean Square	F Ratio
Model	5	57.383173	11.4766	517.8236
Error	231	5.119702	0.0222	Prob > F
C. Total	236	62.502875		<.0001*

Sorted Parameter Estimates

Term	Estimate	Std Error	t Ratio	Prob>|t|
Screw speed (rpm)	-0.008638	0.000249	-34.71	<.0001*
Feed rate (g/min)	0.238805	0.011206	21.31	<.0001*
(Feed rate (g/min)-7)*(Feed rate (g/min)-7)	0.0444055	0.004984	8.91	<.0001*
(Screw speed (rpm)-201.266)*(Screw speed (rpm)-201.266)*(Feed rate (g/min)-7)	-3.863e-6	5.438e-6	-0.71	0.4782
(Screw speed (rpm)-201.266)*(Screw speed (rpm)-201.266)	5.0954e-6	8.056e-6	0.63	0.5277

Prediction Profiler
